# Evaluation of safety and analgesic consumption in patients with advanced cancer treated with zoledronic acid

**DOI:** 10.2478/raon-2013-0041

**Published:** 2013-07-30

**Authors:** Andrej Kmetec, Tine Hajdinjak

**Affiliations:** 1 Department of Urology, University Medical Centre Ljubljana and Medical Faculty Ljubljana, Slovenia; 2 General Hospital Murska Sobota and Medical Faculty Maribor, Slovenia

**Keywords:** diphosphonates, pain, neoplasm metastasis, bone, clinical trial, phase IV

## Abstract

**Background:**

The aim of the study was evaluation of zoledronic acid with regard to safety, effect on analgesic consumption and impact on occurrence of skeletal related events in patients with bone lesions from solid tumors and multiple myeloma.

**Methods:**

We conducted an observational, 12-month, phase IV and multi-center study. One hundred and twenty-five symptomatic (pain) bone-metastatic patients were included between 2007 and 2009: 92 prostate cancers, 28 multiple myelomas, 5 others. They were prescribed monthly infusions of zoledronic acid in accordance to each disease’s treatment guidelines. Analgesics consumption, pain and laboratory values were evaluated.

**Results:**

Zoledronic acid was prescribed concurrent to initial therapy for myeloma and only in late stage of prostate cancer. With treatment, percentage of patients on analgesics decreased in myeloma group (from 57% to 24%) and increased in prostate cancer group (from 70% to 88%). In patients with any analgesics, the use of opiates’ prescription dropped from 72.9% to 64%, percentages of non-steroidal analgesics and other mild analgesics increased slightly. Pain score (Visual Analog Scale, VAS) decreased non significantly (by 22%) in prostate cancer but significantly in myeloma (by 97%). Hypocalcaemia grade 3 or 4 was observed in 4% of patients. Deviations in creatinine remained stable throughout. A total of 31 skeletal related events were reported for 10 patients (8%).

**Conclusions:**

Zoledronic acid was safe medication. Different response of pain was seen between prostate cancer and myeloma patients, which might be due to different stages of disease where it was prescribed according to present guidelines. Possibility of earlier start of treatment should be explored in prostate cancer.

## Introduction

In patients with advanced cancer, bone metastases are a frequent occurrence. Pathological fracture and bone pain may be the first symptoms of the disease. In advanced prostate cancer, bone metastases occur in 74%, sometimes only several years after treatment, causing skeletal complications, pain, impaired mobility, increasing use of analgesics, all of which have a severe impact on the quality of life and survival of the patient.^1^–^3^. Bone pain affects 70% of multiple myeloma patients and is the most common symptom.

At additional risk are patients with advanced hormone sensitive cancers (most commonly prostate cancer, but also some gynaecological cancers) undergoing regular hormonal deprivation therapy. The therapy causes a decrease in bone mineral density and increases the risk of skeletal related events, particularly pathological fractures or spinal cord compression.^2^,^4^ Further, although bone density loss is part of the aging process, cancer patients are exposed to additional risk from cancer affecting bone metabolism, impairing patient mobility and causing calcium and vitamin D deficiency.^5^

To limit described damage from bone involvement of cancer, bisphosphonate treatment with its potential of inhibiting osteoclast activity and bone resorption in both osteolytic and osteoblastic bone metastases has evolved and was extensively justified for different cancers. For example, use of zoledronic acid in prostate cancer was established by work of Saad *et al*.^6^, who in a 24-month placebo-controlled study confirmed that zoledronic acid reduced by 36% the incidence of all forms of skeletal related events. Moreover, the pain scores on a 10-level pain scale diminished by more than 2 points. The study revealed that the drug was effective even in cases when the patient had experienced a pathological bone fracture before the onset of treatment, reducing the risk of second or subsequent skeletal related events. When a skeletal related event occurs during zoledronic acid therapy, this is considered treatment failure by many urologists, and, consequently, the therapy is discontinued. However, literature reveals that continuation treatment nevertheless maintain its efficacy, preventing subsequent skeletal related events.^6^,^7^ Furthermore, the pre-clinical studies demonstrated bisphosphonates’ inhibiting effect on cancer development.^8^ Some showed even increased survival in patients on regular treatment with zoledronic acid compared to the placebo group.^7^

After such extensive research work, bisphosphonates were included into treatment guidelines for different cancers.^9^,^10^ Zoledronic acid is the only bisphosphonate to date demonstrating a statistically significant effect in reducing and delaying the time to first skeletal related event, and reducing the pain due to bone metastases in advanced prostate cancer.

As bisphosphonates are becoming every-day prescribed drug in all cancer treatment settings, it seems useful to verify results from laboratory and phase III trials also in the setting of widespread community use. The purpose of the study was evaluation of zoledronic acid treatment in patients with advanced cancer and bone metastases in relation to its safety, its effect on various serum laboratory values, specifically calcium, on analgesic prescription, bone pain and skeletal related events.

## Patients and methods

This phase IV observational clinical study was designed, implemented and reported in accordance with the International Conference on Harmonisation of Technical Requirements for Registration of Pharmaceuticals for Human Use (ICH) which completed Tripartite Guidelines for Good Clinical Practice. The study was also conducted in accordance with the ethical principles laid down in the Declaration of Helsinki and was approved by the National Medical Ethics Committee, approval No 84/07/10.

The inclusion criteria were confirmed cancer (solid tumour) or multiple myeloma, objective evidence of bone metastases or lesions, presence of significant pain at baseline and age above 18 years. The inclusion took place between 2007 and 2009. Included patients received zoledronic acid (Zometa) at a dose of 4 mg once a month (dose could have been reduced to 3 mg according to product guidelines) and were monitored for 12 months. The pain status of the patients was assessed at each visit by using the Visual Analogue Scale (VAS) and recording analgesics drug prescription. Skeletal related events were recorded. As skeletal related events counted: pathological bone fractures (vertebral and non-vertebral), spinal cord compression, surgery to bone, radiotherapy to bone or change in anticancer therapy to palliate bone pain. At each visit, laboratory values were measured (serum creatinine, calcium, haemoglobin, albumins, alkaline phosphatase [ALP], aspartate aminotransferase [ASAT], alanine amino transferase [ALAT], bilirubin and, in prostate cancer patients, prostate-specific antigen [PSA]).

Statistical analysis was performed by using the confidence interval method; the percentage was calculated with a 95% confidence interval. Statistical analysis was performed using R v 2.10.1 (R Foundation for Statistical computing).

## Results

Inclusion criteria were fulfilled by 125 patients (18 females and 107 males), mean age 69.2 years (from 47 to 89). According to cancer type, 92 patients (73.6%) had prostate cancer, 28 (22.4%) multiple myeloma, 3 (2.4%) kidney cancer and 2 (1.6%) other cancers. As the majority of the patients included were those with multiple myeloma and prostate cancer, the statistical analysis results for kidney cancer and other types of cancer were not sufficiently reliable so we focused on analysis of two groups: prostate cancer patients and multiple myeloma patients.

Reason for zoledronic acid prescription was mostly primary prevention of skeletal related events, but also secondary prevention (prescription to patients, who had skeletal related event before prescription).

Eastern Cooperative Oncology Group (ECOG) performance status of patients at the prescription of zoledronic acid is shown in Table 1.

Out of 125 patients who were included in the study, 100 (80%) finished 12 months observation period. Fourteen (11.2%) patients died and 2 (1.6%) stopped treatment due to progression of disease, 3 (2.4%) stopped treatment due to adverse events and 6 (5%) were lost to follow up. According to cancer type, 75% (21/28) of multiple myeloma patients and 82% (75/92) of prostate cancer patients finished study (not statistically significant difference, p=0.065).

### Pain and analgesics use

For analysis of analgesic requirement, prescribed analgesics were grouped into 3 groups: opioid analgesics (tramadol, hydromorphone, oxycodone, transdermal fentanyl, piritramide, morphine sulphate), non-steroidal antiinflammatory drugs (diclofenac, ketoprofen) and others (paracetamol, metamizole, pregabalin). The total number of prescribed analgesics did not change significantly. The patients were prescribed a maximum of 4 different analgesics concomitantly. The most frequently prescribed analgesic was tramadol (weak opioid), followed by non-steroidal anti inflammatory drugs. During the follow up period, the percentage of patients receiving analgesic treatment increased slightly: 68% (85 subjects, CI 59.8%–76.2%) at Visit 1, 71.9% (82 subjects, CI 57.3%–73.9%) at Visit 6 and 75% (75 subjects, CI 66.5%–83.5%) at Visit 12. At Visit 1, out of those who received analgesic treatment, 72.9 % received opioids, 36.5% non-steroid analgesics, and 17.6% other analgesics and antipyretics. At Visit 6, the percentage of opioids dropped to 65.9%, non-steroid analgesics rose to 37.8%, while 20.7% received other analgesics. At Visit 12, 64% received opioids, 38.7% non-steroid analgesics, and 26.7% other analgesics.

In the multiple myeloma patients, the proportion of those without analgesic treatment increased during the follow-up period. At Visit 1, 16 (57.1%, CI 38.8%–75.5%) enrolled patients received analgesics, at Visit 6, the number of participants receiving analgesics was 13 (50%, CI: 30.8%–69.2%), and at the end of the period, there were only 5 (23.8%, CI 5.6%–42.0%) patients with multiple myeloma receiving analgesics. The number of opioid recipients diminished (at Visit 1, 16 out of 16 patients opioids were given, at Visit 6, 11 out of 13 patients with instituted analgesic therapy received opioids, and at Visit 12, only 4 out of 5 patients on analgesic treatment were still given opioids).

**FIGURE 1.**
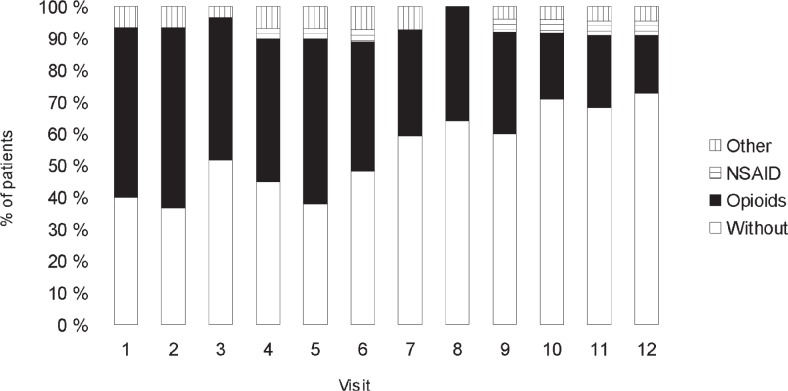
Use of different classes of pain medications by monthly visit in multiple myeloma patients during initial 12-month on zoledronic acid treatment. NSAID = non-steroidal antiinflammatory drugs

In the group of treatment recipients, the percentage of those receiving non-steroid anti inflammatory drugs and those on analgesics was on an increase as a result of a drop in the total number of analgesic recipients. Nevertheless, opioids were still the most frequently prescribed group of analgesics in patients on pain therapy. Analgesics requirements of multiple myeloma patients according to visit are shown in Figure 1.

**FIGURE 2.**
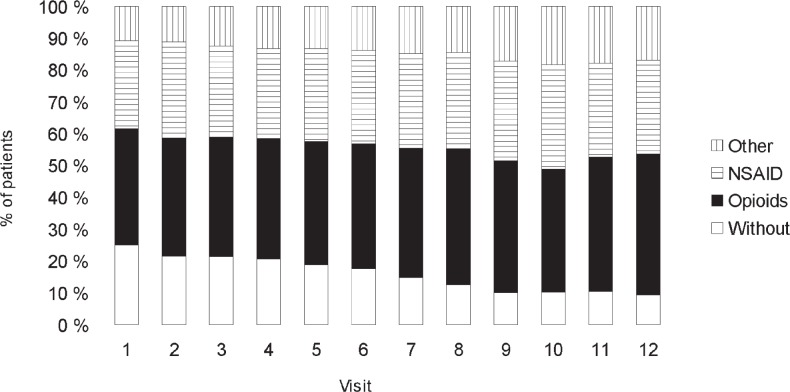
Use of different classes of pain medications by monthly visit in prostate cancer patients during initial 12-month on zoledronic acid treatment. NSAID = non-steroidal anti inflammatory drugs

In the group of prostate carcinoma patients, the percentage of patients without prescribed analgesics dropped during the follow-up period from 30.4 % (CI 21,0%– 39.8%) at Visit 1 to 12 % (CI 4.6%–19.4%) at Visit 12. At Visit 1, analgesic treatment was given to 69.6% of participants (CI 60.2%–79.0%), whereas at the end of the follow-up period, as many as 88% (CI 80.6%–95.4%) of the included patients with prostate carcinoma received analgesics. During the follow up, the percentage of opioid recipients did not vary significantly (at Visit 1, it was 64.1 % (CI 52.3%–75.8%), at Visit 6, it was 60.6 % (CI 48.8%–72.4%), and at Visit 12, it amounted to 63.6 % (CI 52.0%–75.2%). During the follow-up period, the proportion of patients receiving nonsteroidal anti inflammatory drugs diminished: at Visit 1, 31 out of 64 patients with instituted pain therapy, i.e. 48.4% (CI 36.2%–60.7%), at Visit 6, 30 out of 66 patients with pain therapy, *i.e.* 45.5% (CI 33.4%–57.5%) and 28 out of 66 patients, *i.e*. 42.4% (CI 30.5%–54.3%) at the last visit. Analgesics prescription in prostate cancer patients according to visit is shown in Figure 2.

**FIGURE 3.**
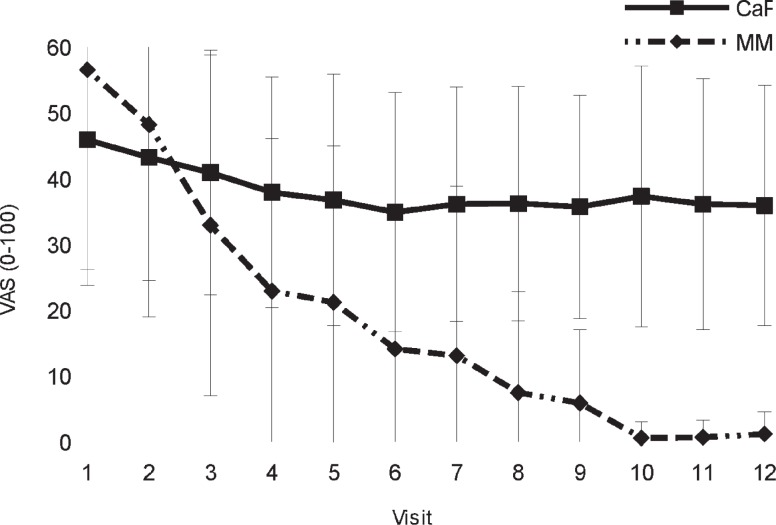
Visual analogue scale pains scores in prostate cancer and multiple myeloma patients during initial 12-month on zoledronic acid treatment. CaF = prostate cancer; MM = multiple myeloma

In multiple myeloma patients, pain sensation diminished during the follow-up, whereas in patients with prostate carcinoma, no significant changes were observed. In kidney cancer patients, VAS score decreased after Visit 5. Graph of mean VAS score and standard deviation according in prostate cancer and multiple myeloma patients during 12 months of study is shown in Figure 3.

### Skeletal related events

During one year of zoledronic acid treatment, new skeletal related events (31 events) were recorded in 10 patients (8%, CI 3.2%–12.8%): 2 with multiple myeloma, 6 with prostate cancer and 2 with other cancers. During the observation period 93.5% of prostate cancer patients and 92.8% of multiple myeloma patients did not experience skeletal related events.

Bone fracture was reported for one patient with multiple myeloma and for 3 patients with prostate cancer. Two bone fractures experienced by one patient with other carcinoma Spinal cord compression was reported for 4 prostate cancer patients, one patient with kidney cancer and one patient with other carcinoma. Bone radiotherapy was performed for one multiple myeloma and one prostate cancer patient. Bone surgery was performed for 3 prostate cancer and one multiple myeloma patients. Change in treatment was related to event in one patient. No cases of hypercalcaemia of malignancy were reported.

### Safety

At least one potential adverse event was reported for 101 patients (80.8 %, CI 73.9%–87.0%). One or more potential adverse events were reported for 18 multiple myeloma patients (64.3%, CI 46.5%–82.0%), 79 prostate cancer patients (85.9 %, CI 78.8%–93.0%) and 4 of 5 other cancer patients (3 patients with kidney cancer, 1 patient with unknown primary). Among very common adverse events, increased serum creatinine was observed in 17.9% multiple myeloma and 32.6% prostate cancer patients. There were 4 occasions of creatinine increase Grade 3 or 4.

**FIGURE 4.**
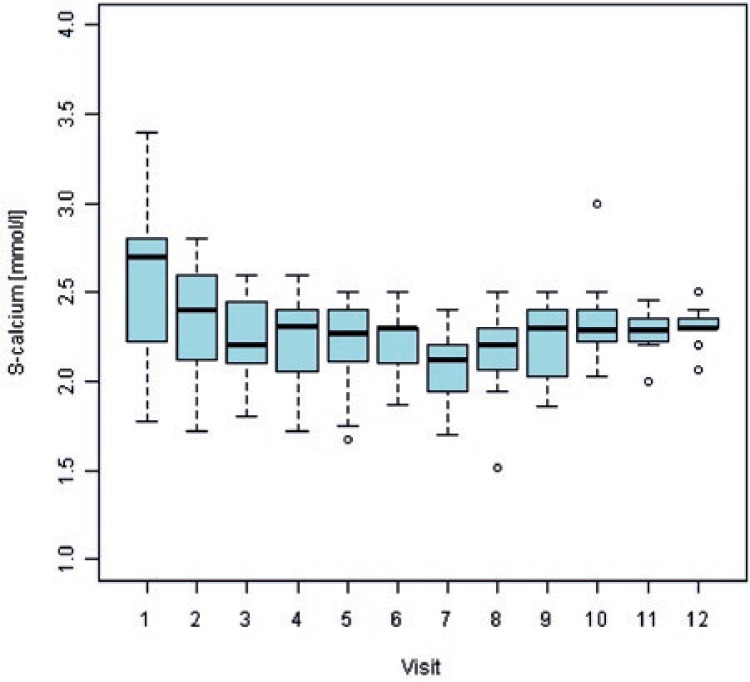
Mean values and variability of serum calcium concentrations by monthly visit from the beginning of zoledronic acid treatment in multiple myeloma patients. S = serum

**FIGURE 5.**
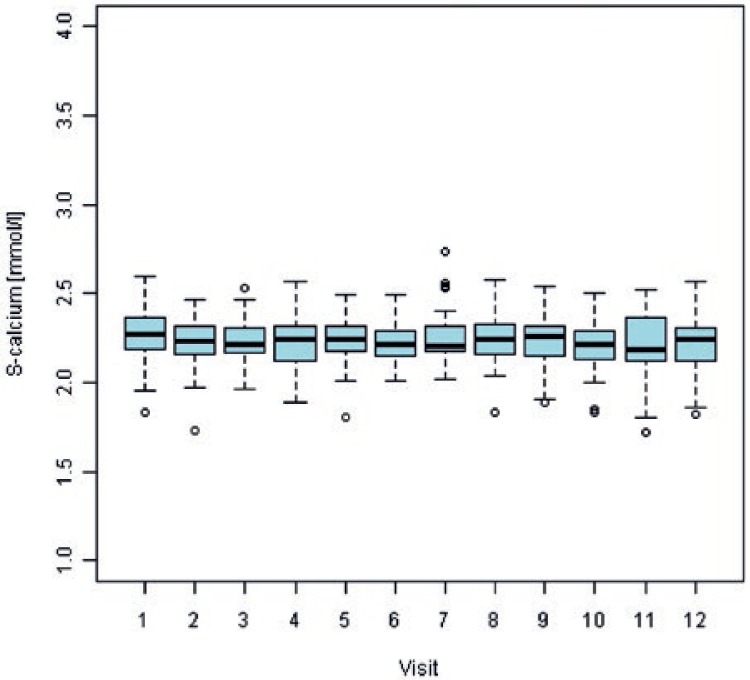
Mean values and variability of serum calcium concentrations by monthly visit from the beginning of zoledronic acid treatment in prostate cancer patients. S = serum

At first visit, 54% of multiple myeloma patients and 3.5% of prostate cancer patients had abnormal (increased) serum calcium values. For multiple myeloma patients, serum calcium values normalized (decreased) in all patients with available data at visit 10 and remained within normal ranges till the end of observation period. For prostate cancer patients, proportion of abnormally low serum calcium values increased during the study period and reached 16.7% at the end of observation period. Overall, hypocalcaemia was observed in 17.9% multiple myeloma patients and in 18.5% prostate cancer patients. There were 5 occasions (5/125; 4%) of hypocalcaemia Grade 3 or 4. Figures 4 and 5 show box plots of serum calcium values by visit for multiple myeloma and prostate cancer patients, respectively.

None of the patients experienced osteonecrosis of the jaw.

Other adverse events (anaemia, elevated PSA) were mainly associated with the primary disease and most cannot be directly linked to zoledronic acid therapy.

## Discussion

Patients with bone metastases experience pain and zoledronic acid treatment is aimed also at improving pain control, which was monitored by recording analgesic prescription pattern. In individual patient groups, the most significant reduction in the overall use of analgesics was recorded for the multiple myeloma group, *i.e*. from 57.1% to 23.8%. Accordingly, sensation of pain diminished in multiple myeloma patients, the reduction being more significant than in other types of carcinoma with bone metastases. The results showed well known fact that with the beginning of treatment, analgesics use and pain scores decrease in multiple myeloma patients. This is observed also in multiple myeloma patients, who do not receive zoledronic acid treatment, but at a lesser extent compared to patients who receive treatment.^11^ Without bisphosphonate treatment 31%–76% pain amelioration is expected.^12^ In our series of multiple myeloma patients, treated with zoledronic acid, much more prominent reduction of pain was observed – 97% if looking at VAS score, mean score decreased from 56.5 (SD 32.8) to 1.2 (SD 3.4). However, this takes into account only patients who remained in trial. In prostate cancer patients, only stabilization of pain score, non-significant reduction of VAS score for 22% was observed (from 46 to 36). In prostate cancer, the percentage of patients requiring analgesic treatment increased from 67% to 88%. The share of opioids remained mostly unchanged. A slight decrease was recorded in the use of non-steroidal analgesics, whereas the share of other, additionally prescribed analgesics increased. Regarding percentage of patients without analgesics, it increased in accordance to decrease in VAS score in group of multiple myeloma patients from 43% to 76%. Percentage of patients without analgesics in prostate cancer group decreased from 33% to 12%, which negates observed (although not significant) decrease in VAS score.

Comparing responses to pain for multiple myeloma and prostate cancer, although obviously different diseases have different progression, it stands out that in multiple myeloma, bisphosphonate treatment is introduced much earlier in the course of disease, synchronously with primary treatment for all patients, irrespectively of bone mineral density, calculations and predictions of future skeletal-related events or confirmation of bone lesions.^10^ In prostate cancer patients, bisphosphonates treatment was indicated only after confirmation of bone lesions and at the same time presence of hormone-refractory disease.^13^ This may be much too late in the progression of disease. If approached similarly to multiple myeloma, all patients who are treated systemically (hormonally) for prostate cancer would be treated with bisphosphonates, perhaps sparing only patients on watchful waiting, which is equivalent disease stage to smouldering myeloma.

Some older prostate cancer patients (older than 70 year of age with lower body mass index) can get low-dose bisphosphonate treatment without bone metastases, if receive androgen deprivation therapy. At this age and taking into account secondary osteoporosis as risk factor, Fracture Risk Assessment Tool (FRAX) calculator for most shows 3% 10 year risk of hip fracture.^13^ Effects of this prophylactic treatment have not been widely studied and should be explored in the future.

The laboratory values for multiple myeloma patients were characterized by a decrease in the percentage of patients with abnormal serum calcium and haemoglobin values, which can be attributed to successful treatment of primary disease as well as to the effect of zoledronic acid. Alkaline phosphatase value in prostate cancer patients remained mostly unchanged throughout the follow-up period. Low haemoglobin values were observed often. In prostate cancer, bone metastases are most frequently found in flat bones which contain bone marrow. Consequently, these patients are diagnosed with anaemia. Among patients with prostate cancer, PSA values were above 4 at the beginning of the study in 78.6% of included patients – this value decreased to 73.6% after one year (of course excluding drop-outs), which cannot be attributed merely to the specific cancer therapy but also to zoledronic acid treatment.

Zoledronic acid is considered safer compared to newer drug for prevention of skeletal related events in cancer patients, denosumab, in regard to frequency of severe hypocalcaemia (grade 3 or 4 according to Common Toxicity Criteria [CTC]).^14^ We observed 4% of grade 3 or 4 hypocalcaemia. This result is in line with literature reports - 4.9% of hypocalcaemia grade 3 or 4 was reported in recent meta-analysis for zoledronic acid treated patients, significantly less than for denosumab, a new drug, with comparable therapeutic effect and with less nephrotoxicity.^15^,^16^ Regarding all measurements of serum calcium below normal limits, we observed 16.8% grade 1 and 2 hypocalcaemias at final visit in prostate cancer patients and 0% at final visit in multiple myeloma patients. For prostate cancer patients, higher percentage of measured low grade hypocalcaemia may be attributed also to less attention to need for monitoring and correcting serum calcium (with enough supplementary calcium and vitamin D) by urologists, who were prescribing zoledronic acid to prostate cancer patients compared to haematologists, who were prescribing zoledronic acid to multiple myeloma patients. In the future, as denosumab is becoming one of opinions for prevention of skeletal related events in prostate cancer patients and as denosumab is known to have twice higher rate of hypocalcaemia compared to zoledronic acid, careful monitoring of serum calcium will become even more important.^16^ On the other side, at the time of writing, this seems not to be a problem for multiple myeloma patients, where denusomab is not approved due to observed lower survival compared to zoledronic acid according to one trial^17^ and remains for this indication strictly investigational.

Zoledronic acid reduces all types of skeletal related events compared to placebo in cancers, metastatic to bone, by 36%.^7^,^8^ Prostate cancer patients are at an even greater risk of skeletal related events than other cancer patients with bone metastases due to hormonal treatment induced secondary osteoporosis. Bisphosphonates were confirmed in many trials to help also in this regard.^18^ In our study, during the observation period, skeletal related events were identified in 8% of the patients (10/125). Trial, similar to ours, which included US prostate cancer patients, reported 11.9% skeletal-related events^19^, which is within the confidence interval of our observation (3.2%–12.8%).

Frequency of suspected adverse events reported (80.8%) is also similar to the observation in US population, where 84% of suspected adverse events were reported.^19^ Adverse events were mainly associated with the primary disease and most cannot be directly linked to zoledronic acid therapy. PSA in prostate cancer patients as a group remained unchanged or even decreased during the observation period which may indirectly support reports of zoledronic acid anticancer activity.^20^,^21^

Limitation of this observational study is lack of the comparator group receiving no zoledronic acid, however, we believe that this would be ethically inappropriate for the time being.

Similar rate of skeletal related events and of potential adverse events in studies from different parts of the world indicate safe and consistent adverse events profile of zoledronic acid. Zoledronic acid is prescribed in different malignancies in different stages of disease: for multiple myeloma at an early stage and for prostate cancer at a late stage of disease, which correlates with pain response, which is much more favourable when drug is prescribed at earlier stage of disease. Further work should explore potential, efficiency and usefulness of zoledronic acid prescription in earlier phase of disease also in solid tumours, specifically those, where it is already used for secondary osteoporosis treatment.

## Figures and Tables

**TABLE 1.** Eastern Cooperative Oncology Group (ECOG) performance status of patients before prescription of zoledronic acid

**ECOG**	**Multiple myeloma N (%)**	**Prostate cancer N (%)**	**Other N (%)**	**Total N (%)**
0	4 (14)	24 (26%)	1 (20)	29 (23)
1	5 (18)	47 (51)	0	52 (42)
2	5 (18)	18 (20)	3 (60)	26 (21)
3	12 (43)	2 (2)	1 (20)	15 (12)
4	2 (7)	1 (1)	0	3 (2)
